# The effect of incorporating an arterial pH target during apnea test for brain death determination

**DOI:** 10.1186/s40560-020-00522-8

**Published:** 2021-01-20

**Authors:** Ibrahim Migdady, Moein Amin, Aaron Shoskes, Catherine Hassett, Sung-Min Cho, Pravin George, Alexander Rae-Grant

**Affiliations:** 1 Division of Neurocritical Care, Department of Neurology, Massachusetts General Hospital, Harvard Medical School, 55 Fruit Street, Lunder 650, Boston, MA 02114 USA; 2 Department of Neurology, Neurological Institute, Cleveland Clinic, Cleveland, OH USA; 3 Departments of Neurology, Neurological Intensive Care, Anesthesiology and Critical Care Medicine, Johns Hopkins University School of Medicine, Baltimore, MD USA; 4 Department of Neurointensive Care, Cerebrovascular Center, Cleveland Clinic, Cleveland, OH USA

**Keywords:** Apnea test, Brain death, pH, Respiratory drive

## Abstract

**Background:**

Persistent apnea despite an adequate rise in arterial pressure of CO_2_ is an essential component of the criteria for brain death (BD) determination. Current guidelines vary regarding the utility of arterial pH changes during the apnea test (AT). We aimed to study the effect of incorporating an arterial pH target < 7.30 during the AT (in addition to the existing PaCO_2_ threshold) on brain death declarations.

**Methods:**

We performed retrospective analysis of consecutive adult patients who were diagnosed with BD and underwent AT at the Cleveland Clinic over the last 10 years. Data regarding baseline and post-AT blood gas analyses were collected and analyzed.

**Results:**

Ninety-eight patients underwent AT in the study period, which was positive in 89 (91%) and inconclusive in 9 (9%) patients. The mean age was 50 years old (standard deviation [SD] 16) and 54 (55%) were female. The most common etiology BD was hypoxic ischemic brain injury (HIBI) due to cardiac arrest (42%). Compared to those with positive AT, patients with inconclusive AT had a higher post-AT pH (7.24 vs 7.17, *p* = 0.01), lower PaO_2_ (47 vs 145, *p* < 0.01), and a lower PaCO_2_ (55 vs 73, *p* = 0.01). Among patients with a positive AT using PaCO_2_ threshold alone, the frequency of patients with post-AT pH < 7.30 was 95% (83/87).

**Conclusion:**

Implementing a BD criteria requiring both arterial pH and PaCO_2_ thresholds reduced the total number of positive ATs; these inconclusive cases would have required longer duration of AT to reach both targets, repeated ATs, or ancillary studies to confirm BD. The impact of this on the overall number BD declarations requires further research.

## Introduction

Brain death (BD), or death by neurological criteria (DNC), is defined by the American Academy of Neurology (AAN) as the cessation of all functions of the brain, including the brainstem [1]. The clinical determination of BD as guided by the AAN has to fulfill three core criteria in the absence of confounders: an irreversible coma of a known cause, a clinical examination consistent with loss of all brain function (including the brainstem), and apnea [1]. The apnea test (AT), usually the last step in BD determination, is diagnostic of BD if a rise of partial pressure arterial of CO_2_ (PaCO_2_) more than 60 mm Hg (or 20 mm Hg above baseline) fails to stimulate the respiratory drive. The rise of PaCO_2_ is most commonly induced via disconnecting the patient from the ventilator while maintaining adequate oxygenation by inserting an O_2_ cannula in the endotracheal tube (oxygen diffusion method) or by other methods [1].

There exists a significant variability regarding BD determination in general, particularly when it comes to the details of the AT [2]. For example, although following arterial pH is recommended before and after the AT, guidelines in the USA and the UK depend solely on a PaCO_2_ threshold, with no specific recommendations regarding changes in arterial pH [3]. On the other hand, Canadian and Australian/New Zealand guidelines require certain pH thresholds (pH ≤ 7.28 in Canada and < 7.3 in Australia/New Zealand) at the end of the AT in order to confirm BD, in addition to the PaCO_2_ threshold [4, 5]. Most recently, an international expert panel formulated multiple consensus statements on the recommendations on determination of BD and suggested that the targets during the AT be pH less than 7.30 and PaCO_2_ of at least 60 mm Hg [6].

In this study, we retrospectively studied brain dead patients who underwent AT in order to investigate the effect of incorporating an arterial pH target < 7.30 during the AT for BD determinations by answering 2 key questions: (1) How often is the pH < 7.30 at the conclusion of a positive AT? and (2) how often is the pH < 7.30 at the end of an inconclusive AT with inadequate CO_2_ rise? We secondarily aimed to study the frequency of AT complications and use of ancillary studies.

## Methods

### Study design and setting

This retrospective observational study was approved by the Cleveland Clinic local institutional review board (IRB 20-335). Written informed consent was waived given the retrospective nature of the study. Using International Classification of Diseases codes for BD, we obtained medical records of consecutive adult patients (≥ 18 years old) who were declared brain dead and underwent the AT at the Cleveland Clinic Health System (CCHS) over a 10-year period (January 2010 to January 2020).

At our institution, determination of BD follows the 2010 AAN guidelines and requires the clinical determination of irreversible neurological injury, an examination consistent with coma and absent brainstem reflexes, and a positive AT. Confounders such as severe hypotension, hypothermia, hypoxia, severe acid-base abnormalities, and other severe metabolic or endocrine abnormalities have to be excluded and corrected prior to BD declaration. All physicians who participate in the declaration of DNC must complete competency training (usually via an online training course) prior to evaluating patients for DNC, and competency must be certified at least every 5 years. The institutional policy requires two consistent examinations performed by two DNC-trained physicians or one trained physician doing two exams separated in time, with no defined time mandated between the two examinations. The AT is performed using the oxygen diffusion technique. Patients are preoxygenated on the ventilator 100% FiO_2_ for 10 min after which a baseline arterial blood gas (ABG) is drawn and the patient is disconnected from the ventilator. Oxygen is then supplied through a tracheal cannula connected to 4–6 L per minute (Lpm) oxygen source, and the patient is carefully monitored for respiratory effort and hemodynamic stability for 5–10 min, after which another ABG is drawn. Repeat ABGs can be done every 5 min after that until goal PaCO_2_ is reached if the patient remains hemodynamically stable. If PaCO_2_ rises to above 60 mm Hg (or 20 mm Hg above baseline) and the patient displays no spontaneous respiratory effort during the AT, the AT is considered positive and the patient declared brain dead. Of note, an arterial pH target is not required at our institution. Ancillary studies are mandated only if complete clinical examination (including AT) cannot be performed or interpreted with complete certainty. If ancillary testing is required, the policy does not mandate a specific number of ancillary studies as one confirmatory study is generally considered acceptable according to AAN guidelines; however, more than one study could be performed at the physician’s discretion.

### Study population

Included patients were ≥ 18 years old, had a documented clinical examination consistent with BD along with a known irreversible cause of coma, underwent the AT, and declared brain-dead via a positive AT, ancillary studies, or both. Formal BD assessment had to be clearly documented for inclusion. Patients were excluded if age < 18, BD declared using clinical exam alone, or if BD was declared using ancillary studies without undergoing the AT. Patients were included if they completed an inconclusive or complicated AT only if they underwent ancillary studies confirming BD. After obtaining patients’ charts, we screened all patients for the above and exclusion criteria. Each patient’s chart, including imaging studies, were reviewed independently by two investigators (I.M. and M.A.) prior to inclusion. Disagreements were resolved through consensus.

### Data collection and reporting

Data were gathered on patients’ demographics (age at time of BD, sex), precipitant and primary etiologies of BD, timing of BD declaration, details of clinical examination, presence of confounders, details of AT including ABG changes, use and results of ancillary studies. If performed, we reviewed radiologic reports and imagines of brain computed tomography (CT), magnetic resonance imaging and angiography (MRI, MRA), cerebral digital subtraction angiography (DSA), transcranial Doppler ultrasounds (TCDs), and single photon emission computed tomography (SPECT). Additionally, reports of electroencephalogram (EEG) were reviewed.

Positive AT is defined according to the AAN guidelines as persistent absence of breathing drive despite an increase of PaCO_2_ to > 60 mm Hg or a 20 mm Hg above baseline in the absence of confounders. The AT is considered inconclusive due to the presence of significant confounders, inadequate CO_2_ rise or development of hypoxia, hypotension, or arrhythmia precluding the completion of the AT. A negative AT constitutes a return of spontaneous respirations during the AT [1]. Ancillary studies were defined as tests of cerebral blood flow or bioelectrical activity supporting the diagnosis of BD as recommended by AAN guidelines [1]. An ancillary study was considered positive if the findings were supportive of brain death, negative if findings are not supportive of brain death, and inconclusive if reliable interpretation of the test was not possible, commonly due to technical reasons (such as artifact on EEG or hyperostosis for TCD). A positive EEG for BD demonstrates electrocerebral silence with no non-artifactual electrical activity > 2 microvolts during a 30-min recording; a positive TCD demonstrates small systolic peaks with absent diastolic flow or a reverberating flow pattern in anterior and posterior circulation bilaterally (if no flow is seen on TCD, the test is considered inconclusive); a positive DSA demonstrates absence of blood flow at the level of entry of the carotid and vertebral arteries to the skull; a positive SPECT demonstrates no cerebral isotope uptake on both anterior and lateral views, including the brainstem; a positive CTA similarly demonstrates no opacification of intracranial vessels.

### Data synthesis and outcomes

The primary objective of this study was to investigate the effect of incorporating an arterial pH target < 7.30 during the AT for BD determinations by answer the following two key questions: (1) how often is the pH < 7.30 at the conclusion of a positive AT (defined as PaCO_2_ > 60 or > 20 mm Hg above baseline)? and (2) how often is the pH < 7.30 at the end of an inconclusive AT due to inadequate CO_2_ rise? In order to answer the first question, we divided the number of brain-dead patients with a post-AT pH < 7.30 by the total number of patients who underwent a positive AT according to current AAN guidelines (post-AT PaCO_2_ > 60 mm Hg or > 20 above baseline). To answer the second question, we the divided the number of patients with post-AT pH < 7.30 by the number of patients with an inconclusive AT due inadequate PaCO_2_ rise

We secondarily aimed to report the frequency of AT complications and use of ancillary studies.

### Statistical analysis

Continuous data were expressed as mean ± standard deviation (SD) as the data were normally distributed. Categorical data were expressed as number and percentage. For comparative analysis, we used Student *t* test for continuous parametric data, Mann-Whitney *U* or Wilcoxon tests for continuous nonparametric data, and Fisher’s exact or Pearson’s chi-square for categorical data as appropriate. A two-sided *p* value < 0.05 was considered statistically significant.

## Results

### Patients’ characteristics

**Fig. 1:**
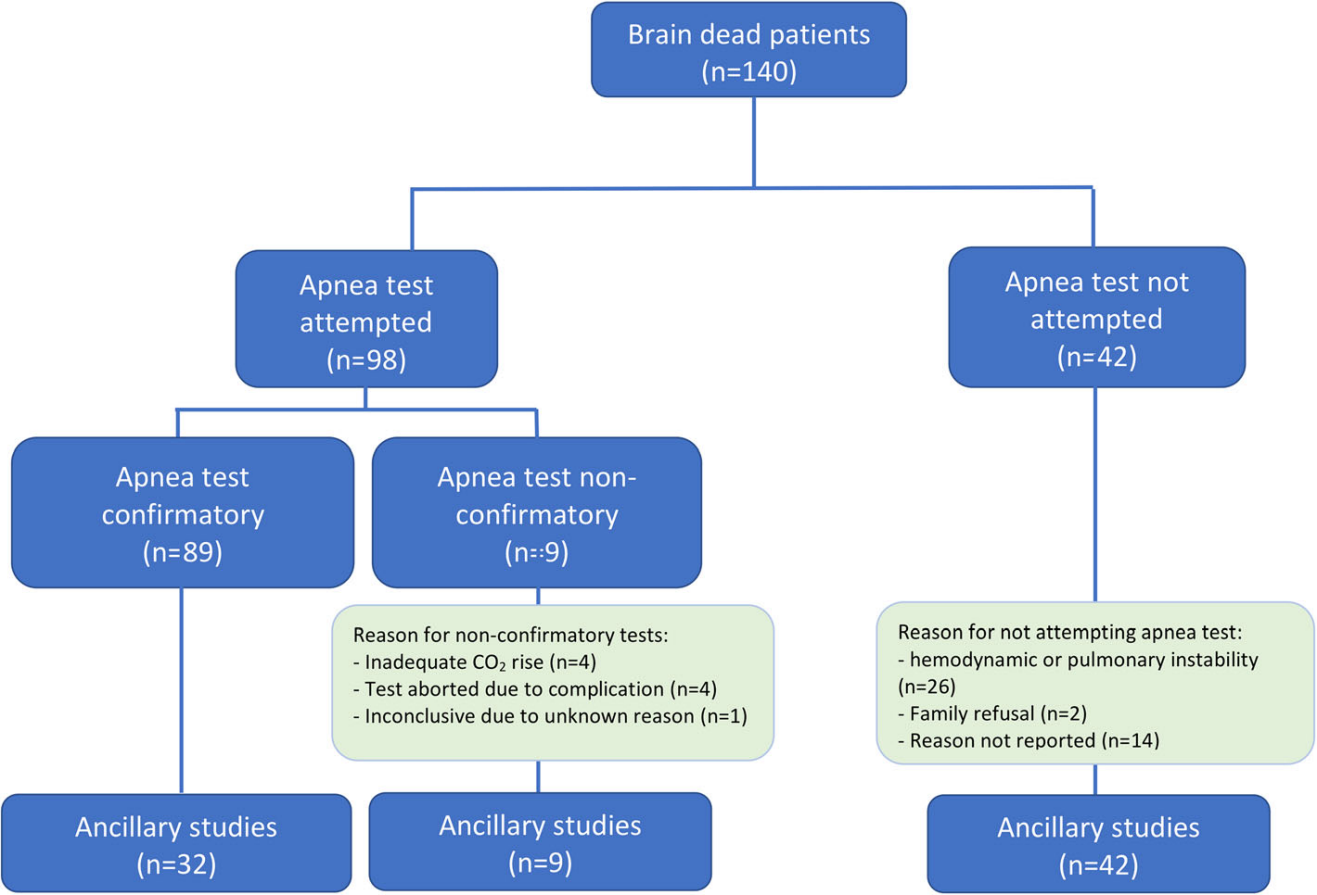
Flow chart of selected patients

Of the 140 patients who were declared brain dead at the Cleveland Clinic Health System between 2010 and 2020, 98 (70%) underwent the AT (meeting our inclusion criteria) and the rest (*n* = 42, 30%) were declared brain-dead using ancillary tests (Fig. 1). The AT was deemed positive in 89 patients (91%) and inconclusive in 9 patients (9%) (Table 1). None of the patients had a negative AT. Among all patients who underwent the AT, the mean age was 50 years old (± 16) and 54 (55%) were female. The primary etiology of BD was hypoxic ischemic brain injury (HIBI) due to cardiac arrest in 41 patients (42%), nontraumatic intracranial hemorrhage in 29 patients (30%), acute ischemic stroke in 11 patients (11%), traumatic brain injury in 9 patients (9%), and cerebral edema due to other causes in 5 patients (5%; 2 due to intracranial malignancy, 1 due to hemodialysis, 1 due to status epilepticus, and 1 due to lupus cerebritis). Three patients (3%) developed cardiac arrest and were found to have evidence of intracranial hemorrhage and later developed HIBI. All patients had two separate clinical examinations confirming coma and absent brainstem reflexes; none of the patients had conflicting clinical exams. The mean interval time between the two exams was 7 h (± 9), which was longer among patients with inconclusive AT compared to those with positive AT (6 vs. 18 h, *p* = 0.03). Reasons for inconclusive AT were inadequate CO_2_ rise in 4 patients (4% of all ATs), inability to complete the AT due to hemodynamic complications in 4 patients (4% of all AT%) and inconclusive results due to unknown reason in 1 patient (1%). Among the 4 patients with inadequate CO_2_ rise, 3 did not tolerate the AT longer than 8–10 min because of hypotension and desaturation; thus, continuing the AT to allow PaCO_2_ to reach target was not attempted; the fourth patient did not achieve adequate PaCO2 rise (41 mm Hg to 51) despite performing the AT for 12 min and a subsequent drop of the pH from 7.32 to 7.23. In the majority of patients, the duration of the AT was documented to be between 8 and 10 min and to follow institutional guidelines, though there was significant difficulty elucidating the actual duration from the charts.

**Table 1:** Characteristics and blood gas changes in 98 patients undergoing apnea test

Variable	All patients (*n* = 98)	Positive AT (*n* = 89)	Inconclusive AT (*n* = 9)	*P* value
Age (years), mean (±SD)	50 (± 16)	50 (± 16)	52 (± 18)	0.80
Female, *n* (%)	54 (55)	47 (53)	7 (89)	0.18
Primary etiology of BD, *n* (%)				
HIBI	41 (42)	35 (40)	6 (67)	0.31
Nontraumatic ICH	29 (30)	29 (33)	1 (11)	0.26
HIBI + Nontraumatic ICH	3 (3)	3 (3)	0	1
AIS	11 (11)	9 (10)	2 (22)	0.27
TBI	9 (9)	9 (10)	0	1
Other cerebral edema	5 (5)	4 (4)	1 (11)	0.39
Malignancy	0	2	0	
Status epilepticus	0	1	0	
Lupus cerebritis	0	0	1	
Hemodialysis	0	1	0	
Duration between clinical exams (hours), mean (±SD)	7 (± 9)	6 (± 9)	18 (± 11)	0.03
Mean BP at the beginning of AT (mm Hg), mean (±SD)				
SBP	127 (± 28)	128 (± 28)	113 (± 31)	0.49
DBP	72 (± 16)	73 (± 16)	65 (± 17)	0.50
MAP	90 ± 18	91 (± 18)	81 (± 21)	0.50
Temperature (°C), mean (±SD)	36.9 ± 0.6	36.9 (± 0.7)	36.9 (± 0.3)	0.79
Vasopressor support, *n* (%)	78 (80)	71 (80)	7 (78)	1
Pre-AT blood gas (mm Hg), mean (±SD)				
pH	7.38 ± 0.06	7.38 ± 0.06	7.37 ± 0.04	0.66
PaO2	242 ± 130	246 ± 130	187 ± 127	0.37
PaCO2	39 ± 5	39 ± 5	37 ± 5	0.37
Post-AT blood gas (mm Hg), mean (±SD)				
pH	7.17 ± 0.07	7.17 ± 0.07	7.24 ± 0.04	0.01
PaO2	234 ± 125	145 ± 120	47 ± 17	< 0.01
PaCO2	72 ± 13	73 ± 12	55 ± 9	0.01
Reasons for inconclusive test, *n* (%)				
Inadequate CO2 rise	4 (4)	NA	4 (44)	
Inability to complete the test due to complication	4 (4)	NA	4 (44)	
Unclear	1 (1)	NA	1 (11)	
Ancillary studies, total *n* (%)	49	38	11	< 0.01
TCD	18 (37)	16 (42)	2 (18)	0.18
SPECT	14 (29)	11 (29)	3 (27)	1
EEG	15 (30)	10 (26)	5 (45)	0.28
CTA	2 (4)	1 (3)	1 (9)	0.40

*AT* apnea test, *SD* standard deviation, *BD* brain death, *HIBI* hypoxic ischemic brain injury, *ICH* intracranial hemorrhage, *AIS* acute ischemic stroke, *TBI* traumatic brain injury, *BP* blood pressure, *SBP* systolic blood pressure, *DBP* diastolic blood pressure, *MAP* mean arterial pressure, *PaO2* partial pressure of arterial O2, *PaCO2* partial pressure of arterial CO2, *TCD* transcranial Doppler, *SPECT* single photon emission computed tomography, *EEG* electroencephalogram, *CTA* computed tomography angiography

### Physiological changes during the AT

Blood pressure values at the beginning of AT were available in 74 patients (75%); mean SBP, DBP, and MAP (mmHg) were 127 (± 28), 72 (± 16), and 90 (± 18), respectively. Body temperature at the time of AT was available in 87 patients (89%) and the mean temperature was 36.9 °C (± 0.6). Seventy-eight patients (80%) were on vasopressor at the time of AT. There was no significant difference in mean SBP, DBP, MAP, temperature, or vasopressor support between patients with positive and inconclusive tests (Table 1).

**Fig. 2:**
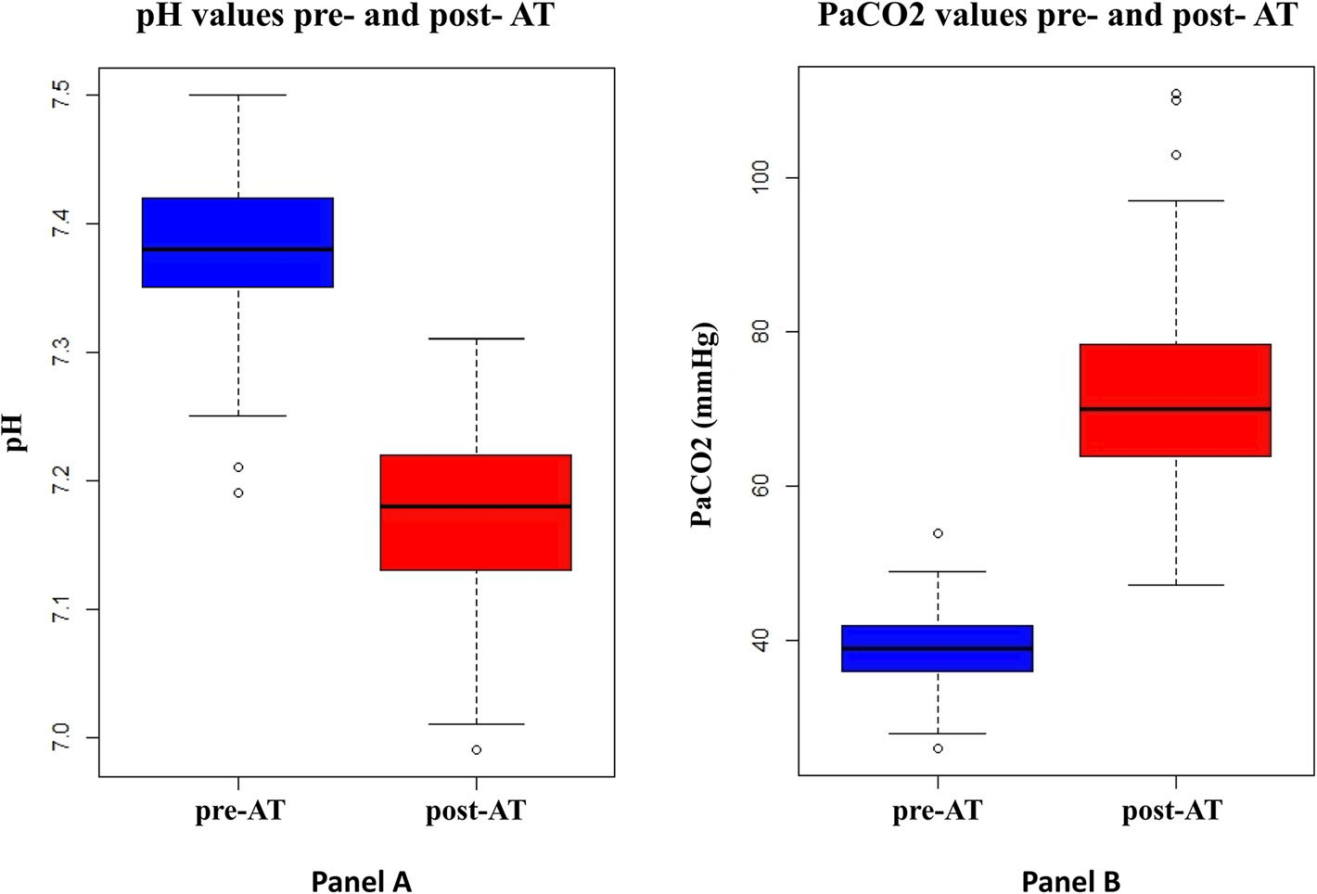
Box plots of PaCO_2_ and pH values before and after the apnea test. **a** pH values pre-AT (median = 7.38, IQR = 0.07) and post-AT (median = 7.18, IQR = 0.09). **b** PaCO_2_ values pre-AT (median = 39.0, IQR = 5.8) and post-AT (median = 70.0, IQR = 14.3). Abbreviations: PaCO_2_, partial pressure of arterial CO_2_; AT, apnea test; IQR, interquartile range

Pre- and post-AT ABGs were available for 91/98 patients (93%). In the remaining 7 patients, the outcome of the AT (2 positive, 4 complicated and 1 inconclusive due to unknown reason) was documented in the chart but not all ABG values were available for review. The mean pre-AT pH, PaO_2_, and PaCO_2_ for all patients were 7.38 (± 0.06), 243 mm Hg (± 130) and 44 mm Hg (± 6) respectively. The mean post-AT pH, PaO_2_, and PaCO_2_ among all patients were 7.17 (± 0.07), 234 mm Hg (± 125), and 72 mm Hg (± 13), respectively (Fig. 2). Compared to those with positive AT, patients with inconclusive AT had a higher post-AT pH (7.24 vs 7.17, *p* = 0.01), lower PaO_2_ (47 vs 145, *p* < 0.01), and a lower PaCO_2_ (55 vs 73, *p* = 0.01) (Table 1).

### Outcomes

Among patients with a positive AT using PaCO_2_ threshold according to current AAN guidelines, the frequency of patients with post-AT pH < 7.30 was 95% (83/87) (Table 2). Among those with an inconclusive test due to inadequate CO_2_ rise, the frequency of patients with post-AT pH < 7.30 was 75% (3/4).

**Table 2:** Relationship between arterial pH and PaCO_2_ at the conclusion of apnea test

Value at end of AT	PaCO2 > 60 or > 20 mm Hg above baseline (positive AT) ^‡^, *n*	PaCO2 < 60 or < 20 mm Hg above baseline (inconclusive AT) ^‡^, *n*
pH < 7.30	83	3
pH ≥ 7.30	4	1
Total	87	4

*AT* apnea test, *BD* brain death

^‡^Seven patients were excluded from analysis (4 due aborted AT due to complication, and 3 due unavailable pH data). Patients with inconclusive AT underwent ancillary studies to confirm BD

Collectively, complications of the AT due to hypoxia, hypotension, or arrhythmia occurred in 9 patients (9%), 4 of which (4% of all ATs) resulted in early termination of AT. Among patients with a positive AT, 32/89 patients (36%) underwent a total of 38 ancillary studies (16 TCD, 11 SPECT, 10 EEG, 1 CTA; 4 patients underwent two ancillary tests). All 9 patients (100%) with inconclusive AT underwent a total of 11 ancillary studies (5 EEG, 3 SPECT, 2 TCD and 1 CTA; 2 patients underwent two ancillary studies).

## Discussion

In this retrospective study involving a large number of patients who underwent AT and eventually diagnosed with BD in compliance with the 2010 AAN guidelines, arterial pH < 7.30 in 95% of those with a positive AT. In other words, implementing new BD guidelines that require both an arterial pH threshold of < 7.30 in addition to PaCO_2_ > 60 or > 20 mm Hg above baseline (as recommended by the World Brain Death Project Consensus Statement) [6] would mean that 5% of patients who were declared BD using AT relying on PaCO_2_ thresholds alone would not immediately be declared BD using clinical criteria if both pH and PaCO_2_ threshold were to be used, and their AT would either need to be repeated, prolonged, or an ancillary study to be used. In other words, if such a criteria had been in place, it is possible that clinicians would perform the AT for a longer period of time until reaching target pH and PaCO2, repeat the AT, or confirm BD with ancillary studies. This may not cause any change in the total number of BD declarations, since those patients may still be diagnosed with BD using a repeated AT or ancillary studies. Interestingly, among 4 patients who were persistently apneic during AT despite an inadequate PaCO_2_ rise, 3 had pH < 7.30, raising the question whether low arterial pH alone can be relied upon to assess respiratory drive; however, the small number patients with this ABG profile limits any overreaching conclusions.

The ABG results in our cohort is consistent with recent studies of ABG changes during AT. In a recent analysis of 114 brain dead patients who underwent the AT at the Mayo Clinic, the pre-AT median arterial pH was 7.38 (interquartile range [IQR] 1.0) the post-AT median pH of 7.18 (IQR 0.09) [7]. An earlier analysis of 63 patients who underwent the apnea showed a pre-AT median arterial pH of 7.36 (IQR 1.0) and post-AT pH of 7.19 (IQR 1.0) [8]. However, these studies have not examined the use of post-AT arterial pH as threshold for confirming apnea. Although the most recent AAN guidelines (in 2010) did not incorporate arterial pH into the criteria for a positive AT, as discussed above, Canadian and Australian/New Zealand guidelines require certain pH thresholds (pH ≤ 7.28 in Canada and < 7.30 in Australia/New Zealand) at the end of the AT in order to confirm BD, in addition to the PaCO_2_ threshold [4, 5]. There have been recent calls for a worldwide standardization of BD determination, including incorporating an arterial pH threshold during the AT for during BD determinations [6].

The utility of arterial pH during AT has a strong physiological basis. The goal of the AT is to stimulate the respiratory drive by increasing PaCO_2_; however, in addition to PaCO_2_, pH has an essential role in stimulating the respiratory drive. First, peripheral chemoreceptors in the carotid and aortic bodies function to detect the PaO_2_ in the blood. Once stimulated by hypoxia, neuronal signals are sent to medullary nuclei to stimulate the respiratory drive and improve oxygenation in a pH-dependent manner (i.e., the sensitivity of peripheral chemoreceptors to low PaO_2_ is increased by acidosis) [9]. Second, the central chemoreceptors in the lower brainstem (medulla) are activated primarily in response to direct effects of local H^+^ on pH-sensing proteins, which changes in response to changes in PaCO_2_ and arterial pH [10]. Due to the enzyme carbonic anhydrase, CO_2_ is in equilibrium with H^+^, and the effect of hypercapnia on the breathing drive is largely mediated through changes in local pH around these pH-sensing neurons [10]. In addition to the effect of hypercapnic acidosis on the respiratory drive, several in vitro studies showed that local “isocapnic acidosis” (low pH in the setting of normal pressure of CO_2_) can result in a similar magnitude of respiratory drive stimulation [11, 12], and in vivo animal studies utilizing microinjections of acetazolamide (a carbonic anhydrase inhibitor inducing local acidosis) into different areas of the brainstem increases ventilation [13, 14]. Similarly, changes in cerebrospinal fluid (CSF) pH from 7.30 to 7.25 were shown to double the rate of alveolar ventilation [15]. One could argue that changes in arterial pH are not necessarily reflected in CSF pH and thus may not directly stimulate the respiratory drive; however, several human studies showed a predictable change in CSF pH compared to arterial pH, resulting in CSF pH ranging between 0.02 and 0.10 units below that of the arterial blood [16]. This concept is the primary mechanism behind the respiratory compensation (manifesting as hyperventilation) in response to metabolic acidosis. However, there are several unanswered questions regarding the use of pH in AT; the lowest pH at which a person has the potential to breathe has not been clearly defined; and changes in arterial pH alone may not reflect fast enough on CSF pH to stimulate the respiratory drive within the duration of the AT. Whether there are clinically brain-dead patients who are apneic at PaCO_2_ > 60 but start breathing when pH is lower than 7.30 (or other suggested targets) has not been well-studied. Additionally, the effect of a pH criterion on AT outcomes in patients on extracorporeal membrane oxygenation remains to be investigated [17].

This study is motivated by a strong physiological basis for using arterial pH to aid in clinical determination of BD, as discussed above. Another strength of the study is the large number of patients who underwent AT, which improves our understanding of ABG changes. The major limitation of our study is its retrospective nature, which renders it prone to recall and misclassification bias although the electronic charts were carefully assessed to ensure BD was diagnosed according to AAN guidelines in order to limit the possibility of bias. The impact of incorporating pH targets during the AT is better assessed prospectively. Additionally, there is a significant variability of BD practice worldwide [6], which limits the generalizability of findings from this single-center study.

## Conclusion

During AT for BD determination, requiring both arterial pH and PaCO_2_ thresholds resulted in 5% decrease in the frequency of patients with positive AT; these cases would have required either longer period of time until reaching target pH and PaCO_2_, repetition of the AT, or confirmation of BD with ancillary studies. The impact of this on the overall number of BD declarations requires further study.

## Data Availability

The data supporting the conclusions of this article are available upon request from the corresponding author upon reasonable request.

## Abbreviations

BD: Brain death
DNC: Death by neurological criteria
AAN: American Academy of Neurology
AT: Apnea test
CCHS: Cleveland Clinic Health System
ABG: Arterial blood gas
CT: Computed tomography
MRI: Magnetic resonance imaging
MRA: Magnetic resonance angiography
DSA: Digital subtraction angiography
TCD: Transcranial Doppler
SPECT: Single photon emission computed tomography
EEG: Electroencephalogram
SD: Standard deviation
SBP: Systolic blood pressure
DBP: Diastolic blood pressure
MAP: Mean arterial pressure
IQR: Interquartile range
CSF: Cerebral spinal fluid
